# A Compact Viral Processing Proteinase/Ubiquitin Hydrolase from the OTU Family

**DOI:** 10.1371/journal.ppat.1003560

**Published:** 2013-08-15

**Authors:** Charlotte Lombardi, Maya Ayach, Lionel Beaurepaire, Mélanie Chenon, Jessica Andreani, Raphaël Guerois, Isabelle Jupin, Stéphane Bressanelli

**Affiliations:** 1 The Laboratoire de Virologie Moléculaire et Structurale, Centre de Recherche de Gif, CNRS (UPR 3296), Gif sur Yvette, France; 2 Laboratoire de Virologie Moléculaire, CNRS - Univ Paris Diderot, Sorbonne Paris Cité, Institut Jacques Monod, UMR 7592, Paris, France; 3 CEA, iBiTecS, Service de Bioénergétique Biologie Structurale et Mécanismes (SB2SM), Laboratoire de Biologie Structurale et Radiobiologie (LBSR), Gif sur Yvette, France; Purdue University, United States of America

## Abstract

Turnip yellow mosaic virus (TYMV) - a member of the alphavirus-like supergroup of viruses - serves as a model system for positive-stranded RNA virus membrane-bound replication. TYMV encodes a precursor replication polyprotein that is processed by the endoproteolytic activity of its internal cysteine proteinase domain (PRO). We recently reported that PRO is actually a multifunctional enzyme with a specific ubiquitin hydrolase (DUB) activity that contributes to viral infectivity. Here, we report the crystal structure of the 150-residue PRO. Strikingly, PRO displays no homology to other processing proteinases from positive-stranded RNA viruses, including that of alphaviruses. Instead, the closest structural homologs of PRO are DUBs from the Ovarian tumor (OTU) family. In the crystal, one molecule's C-terminus inserts into the catalytic cleft of the next, providing a view of the N-terminal product complex in replication polyprotein processing. This allows us to locate the specificity determinants of PRO for its proteinase substrates. In addition to the catalytic cleft, at the exit of which the active site is unusually pared down and solvent-exposed, a key element in molecular recognition by PRO is a lobe N-terminal to the catalytic domain. Docking models and the activities of PRO and PRO mutants in a deubiquitylating assay suggest that this N-terminal lobe is also likely involved in PRO's DUB function. Our data thus establish that DUBs can evolve to specifically hydrolyze both iso- and endopeptide bonds with different sequences. This is achieved by the use of multiple specificity determinants, as recognition of substrate patches distant from the cleavage sites allows a relaxed specificity of PRO at the sites themselves. Our results thus shed light on how such a compact protein achieves a diversity of key functions in viral genome replication and host-pathogen interaction.

## Author Summary

Positive-stranded RNA viruses are ultimate parasites. In order to replicate their genome, they first need to invade a host cell and, with usually very limited viral genetic material, subvert the host's molecular machinery. Turnip yellow mosaic virus (TYMV) is an excellent model system for studying positive-stranded RNA virus replication. As for many such viruses, TYMV genome replication is dependent on the activity of a viral proteinase (PRO) to properly process the virus' replication molecules. We have recently established that PRO is a multifunctional enzyme and is also used by TYMV to subvert a key host defense against pathogens. We report here the atomic structure of PRO as well as new functional data on PRO's interaction with the host. Our data shed light on how PRO can perform such multiple activities despite its small size, providing TYMV with a Swiss army knife in its ongoing fight with a vastly more complex host.

## Introduction

Plus-strand RNA (RNA+) viruses are the largest class of eukaryotic viruses. They include significant pathogens of humans, animals and plants [1]. From the sequencing of their genomes, it has become clear that despite a huge diversity, these viruses possess high similarities at the molecular level [2] [3]. Indeed, common strategies and regulatory mechanisms have been uncovered in the replication of RNA+ viruses [4]. Thus, all RNA+ viruses studied to date synthesize new viral genomes at an intracellular membrane. There, synthesis of the viral progeny requires the establishment of specific and regulated interactions between viral proteins and different cellular factors, assembled within a replication complex. In RNA+ viruses, the replication proteins are usually synthesized as a single polypeptide chain that may be subsequently processed by viral (and sometimes also cellular) proteinases. Another common feature of RNA+ viruses is that the highly compact viral genome codes for usually multifunctional proteins.

Turnip yellow mosaic virus (TYMV) is a simple, model RNA+ virus whose replication is well characterized at the molecular and cellular levels [5] [6] [7]. It is included in the alphavirus-like supergroup of RNA+ viruses [3] that also comprises the animal alphaviruses (including Sindbis virus, Semliki Forest virus and Chikungunya virus) and rubiviruses (including Rubella virus). Indeed, TYMV shares with these viruses striking similarities in the organization and processing of the replication polyprotein [8]. Its 6.3-kb genome codes for three proteins, the largest of which (206K) is a polyprotein of 206 kDa that contains all the viral components of the replication machinery (Fig. S1 in Text S1). From N- to C-terminus, 206K harbors methyltransferase (MT), cysteine proteinase (PRO), helicase (HEL or 42K) and RNA-dependent RNA polymerase (POL or 66K) domains. In previous works, we established that the PRO domain is a key regulator of TYMV replication. First, its endopeptidase activity is required to proteolytically process 206K at the HEL/POL junctions to release the 66K polymerase, while a second cleavage at the PRO/HEL junction contributes to the regulation of viral RNA synthesis [8]. The PRO domain is also essential for the recruitment of 66K to the membrane replication sites [7]. Finally, we recently reported that TYMV PRO also displays an ubiquitin hydrolase (DUB) activity *in vitro* and *in vivo*, and identified 66K polymerase as a specific substrate of this activity [9]. PRO's DUB isopeptidase activity is thus also a key factor for the interaction of the virus with its host, in counteracting the ubiquitin-proteasome system and possibly subverting it into regulating availability of 66K for TYMV replication [10].

Here we describe the crystal structure of the recombinant PRO. Strikingly, PRO displays no homology to other processing proteinases from RNA+ viruses, including that of alphaviruses, and the closest structural homologs of PRO were identified as DUBs from the Ovarian tumor (OTU) family. Our crystal captures a view of TYMV PRO in its polyprotein processing mode that reveals dual substrate specificity determinants. Modelling of a PRO/ubiquitin complex, subsequent site-directed mutagenesis of PRO and enzymatic analysis of its DUB activity suggest that PRO structural elements used for specific recognition of ubiquitin overlap those used in its processing proteinase function. These findings provide a structural rationale for PRO's targeting of the diverse viral and cellular, endo- and isopeptide bonds whose hydrolysis allows TYMV to complete its replication cycle.

## Results

### A three-lobed proteinase with the catalytic domain of a divergent OTU DUB

We report here the structure of the TYMV PRO domain to a resolution of 2 Å with a final *R*_free_ of 20.1% (Table 1). As reported elsewhere [11], all data including 3 derivative datasets obtained by heavy atom soaks were from crystals grown in a single crystallization drop. The asymmetric unit contains a single PRO molecule packing against the next PRO along the crystallographic 3_1_ screw axis, making up continuous PRO helices in the crystal (Fig. S2 in Text S1), and an *Escherichia coli* contaminant (ribosomal protein S15). S15 bridges the separate PRO helices, explaining why diffraction-quality crystals only grew from a PRO preparation heavily contaminated by S15 [11].

**Table 1:** Data processing statistics and final structure refinement and model validation.

Diffraction source	SOLEIL - PX1
Wavelength (Å)	0.8856
Temperature (K)	100
Space group	*P*3_1_21
Cell parameters (Å)	*a* = 135.2, *c* = 41.9
Resolution range (Å)	39.5–2.000 (2.05–2.00)
No. of unique reflections	29610 (2175)
Completeness (%)	99.04 (99.5)
Redundancy	3.72 (3.74)
<I/s(I)>	14.8 (2.2)
R_sym_	6.4 (61.2)
Data-processing software	XDS XSCALE
Refinement software	phenix.refine
Refinement on	F
s cutoff	none
Resolution range (Å)	39.029–2.000 (2.07–2.00)
No. of reflections used in refinement	29610 (2719)
Atomic displacement model	TLS+restrained individual B factor
Overall average B factor (Å^2^)	33.23
Final R_work_	0.1678 (0.2224)
No. of reflections for R_free_	2275 (220)
Final R_free_	0.2011 (0.2769)
Average RMSD Bond Angles	1.205
Average RMSD Bond Lengths	0.008
Ramachandran plot analysis	
Favoured regions (%)	99.12
Allowed regions (%)	0.88
Outliers regions (%)	0

Values for the outer shell are given in parentheses.

R_sym_ was determined by the equation inline-formula 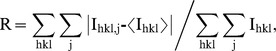 where h, k and l are the unique indices of all reflections measured more than once and j the index for symmetry-redundant reflections.

R and R_free_ were determined by the equation 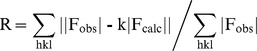 where h, k and l are the indices of the reflections used in refinement (R) or the reflections set aside and not used in refinement (R_free_). F_obs_ are the structure factors deduced from the measured intensities and F_calc_ the structure factors calculated from the model. k is a scale factor to put the F_calc_ on the same scale as the F_obs_.

**Figure 1:**
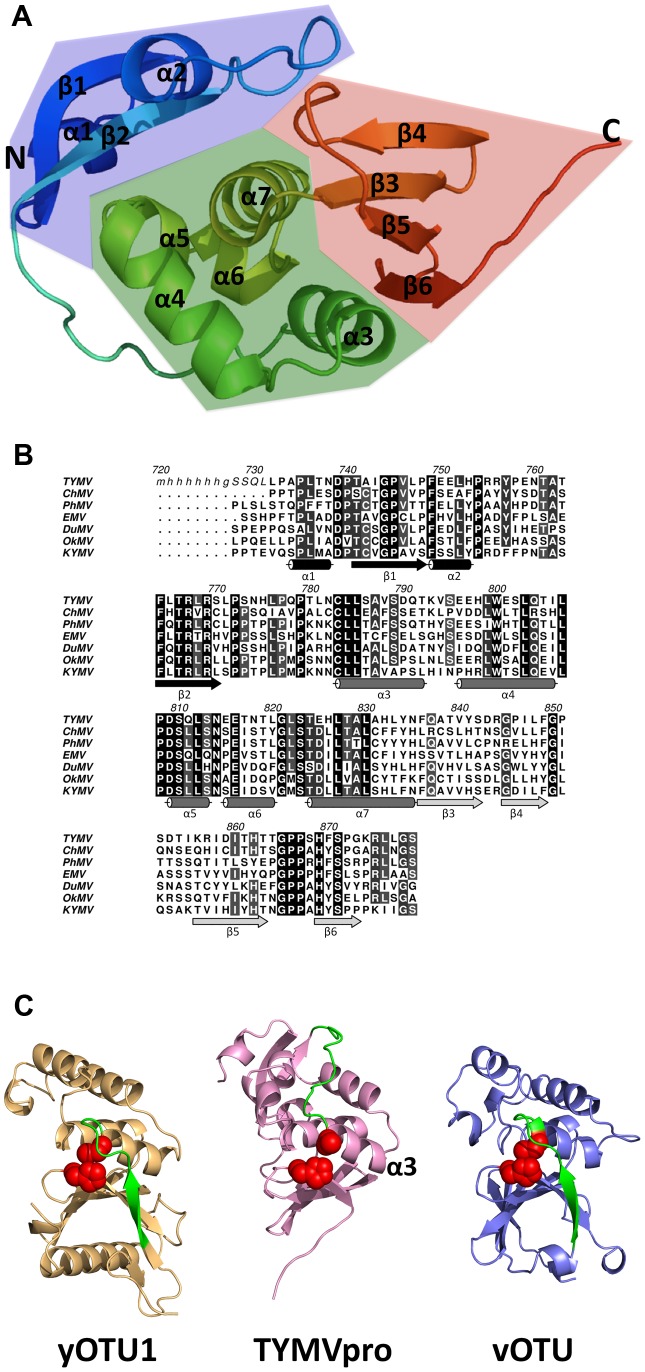
Overall structure of PRO and comparison with its closest relatives. A, Organization of PRO in three lobes displayed as ribbons. The N-terminal lobe is colored in blue, the central lobe in green and the C-terminal lobe in red. Secondary structure elements are labeled: α for helices and β for β-sheets with numbers from N to C-terminal for each kind of element. B, Amino acid sequence alignment of polyprotein processing endopeptidases belonging to the Tymoviridae family. Proteinase domain sequences are displayed as capital letters for TYMV (Turnip yellow mosaic virus), ChMV (Chayote mosaic virus), PhyMV (Physalis mottle virus), EMV (Eggplant mosaic virus), DuMV (Dulcamara mottle virus), OkMV (Okra mosaic virus) and KYMV (Kennedya yellow mosaic virus). Alignment was obtained from the MEROPS database (http://merops.sanger.ac.uk) and edited and displayed using the ALINE program [36]. The numbering of TYMV polyprotein is used here. Italics in the TYMV PRO sequence indicate the N-terminal part of the crystallized construct not visible in the electron density. Lower case letters denote the hexahistidine tag added for purification. The secondary structure of PRO is reported below the alignment. Structural elements belonging to the N-terminal lobe appear in black, those in the central lobe appear in gray and finally those in the C-terminal lobe in light gray. C, The three OTU DUBs TYMV PRO, yOTU1 (cellular OTU DUB, PDB code 3BY4) and vOTU (Bunyaviridae OTU DUB, PDB code 3PRP) are shown as ribbons without their substrates and in the same orientation after superposition by DALI. They are colored pink (PRO, middle panel), light orange (yOTU1, left panel) and light blue (vOTU, right panel). The segments leading to the catalytic cysteine are in green. The catalytic cysteine and histidine are displayed as red spheres.

PRO displays a three-lobed architecture. The N-terminal lobe (in blue on Fig. 1A) comprises two short helices flanking a two-stranded, distorted β-sheet. The catalytic domain is made up by the central and C-terminal lobes (a bundle of five helices and a four-stranded β-sheet, respectively). The catalytic dyad Cys783-His869 (TYMV polyprotein numbering) lies at the interface between helix α3 (the first helix in the central lobe) and strand β6 (the last strand of the C-terminal lobe). Indeed, Cys783 is the first residue of helix α3 and His869 the first residue of strand β6 (Fig. 1B). We used the DALI server [12] (http://ekhidna.biocenter.helsinki.fi/dali_server) to seek homologs of PRO with available structures in the Protein Data Bank (PDB). Strikingly, there is no detectable homology (DALI Z-score below 2) to other processing proteinases from RNA+ viruses, including that of alphaviruses. It was previously remarked that PRO shares limited sequence similarities around the two catalytic residues with the OTU domain class of DUB enzymes and we recently reported that PRO is a functional DUB *in vitro* and *in vivo* [9]. Indeed, although no close homolog is available and the N-terminal lobe cannot be matched at all, the fold of the PRO catalytic domain is clearly the same as the core fold of the OTU1 cellular DUB (yOTU1, *Saccharomyces cerevisiae*, DALI Z-score 7.5, 91 residues matched) [13] and nairovirus DUB (vOTU, *Bunyaviridae*, DALI Z-score 6.8, 90 residues matched) [14] [15] [16]. These two DUBs are assigned to clan CA of papain-like proteinases in the MEROPS peptidase database scheme [17] (http://merops.sanger.ac.uk/). Although clan CA contains several viral processing proteinases from *Picornaviridae* and *Coronaviridae*, only strict DUBs (*i.e.* enzymes lacking endopeptidase activity) have substantial DALI Z-scores in comparisons with PRO. Indeed, the nearest homolog of PRO with reported endopeptidase activity is the bacterial Staphopain (Z-score 3.4).

### An exposed, pared down active site

**Figure 2:**
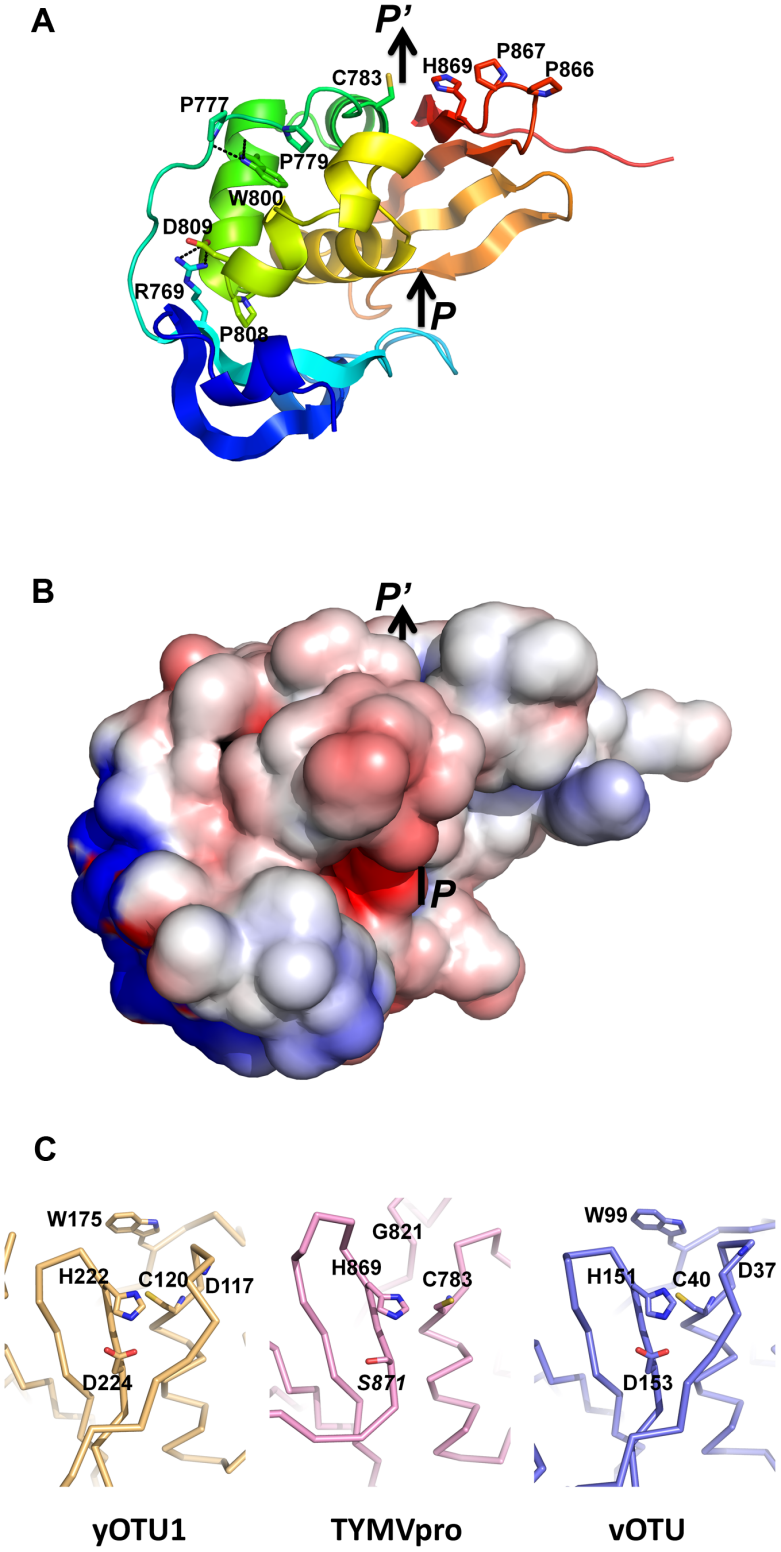
Surface features and active site of PRO. In panels *A* and *B*, the entry to and exit from the catalytic cleft are marked with arrows labeled *P* and *P*', respectively. A, Exposure of the catalytic dyad at the exit from the active site. PRO is displayed in ribbon representation and colored from blue (N-terminus) to red (C-terminus). Residues that are labeled and displayed as sticks with nitrogens in blue, oxygens in red and sulfur in yellow are the catalytic dyad (Cys783-His869), the double cis-proline (Pro866-Pro867) and residues central to the positioning of the N-terminal lobe on the other side from the catalytic dyad. For the latter, important hydrogen bonds and salt bridges are indicated by black dotted lines. B, Electrostatic potential at the surface of PRO. Orientation is the same as in A. Note the apolar bulge of the double cis-proline, the acidic pocket next to the entry to the catalytic cleft, and the basic patch on the outer surface of the N-terminal lobe. C, The active site of PRO (middle) compared to those of yOTU1 and vOTU. View from the exit of the catalytic clefts. The three active sites have been superimposed on the atoms of the catalytic Cys and His except for the Cys sulphur (rmsd of 0.35–0.45 Å for 15 atoms). The side chains of the catalytic Cys and His are displayed as sticks for the three peptidases. The side chain of the putative catalytic aspartate is also displayed for vOTU and yOTU1, as well as the corresponding Ser871 in PRO. The backbone amide nitrogen and the tryptophan side chain that are part of the oxyanion hole in vOTU (D37, W99) and likely yOTU1 (D117, W175) are also displayed.

A DALI superposition of yOTU1 and vOTU yields a Z-score of 12.2. This higher score is due to yOTU1 and vOTU being structurally superimposable on a significantly larger number of residues (126 residues matched by DALI). Of note, in both yOTU1 and vOTU, the segment directly upstream of the homolog of helix α3 (in green on Fig. 1C) partially covers the exit from the active site. In contrast, the catalytic dyad of TYMV PRO Cys783-His869 is completely solvent exposed (Figs. 1C, 2A and 2C). There is no pocket that could act as a stabilizer for the oxyanion intermediate in the reaction for cysteine and serine proteases. Indeed, due to the lack of a covering segment there is no counterpart for the main chain nitrogen of Asp37 of vOTU (Fig. 2C), that has been proposed to participate in formation of this oxyanion hole [15]. Furthermore, the side chain of Trp99 of vOTU, that has been shown to take part in oxyanion hole formation [16], is missing in TYMV PRO's Gly821 (Fig. 2C). Similarly, there is no candidate in PRO for a catalytic residue acting as a general acid to stabilize and activate the side chain of His869. Asp153 of vOTU, that has been shown to be implicated in the catalytic triad [16], is replaced by a serine in *Tymoviridae* as Trp99 is replaced by a glycine (Fig. 2C, Fig. 1B). Thus, TYMV PRO's catalytic site appears to be reduced to an exposed dyad, possibly explaining in part its poor DUB activity [9] (see below) compared to *e.g.* vOTU [15] [14] [16]. Furthermore, although the dyad itself is almost superimposable with the corresponding residues of yOTU1 and vOTU (Fig. 2C and legend thereof), the Cys783 side chain is flipped and makes no interaction with the His869 side chain. Thus the PRO active site is most likely not in its catalytically competent state in the crystal, where we caught a product release state (see below).

### A distal N-terminal lobe and some remarkable surface features

The PRO active site's exposure is due to the long β2–α3 loop (residues 771–782) connecting the N-terminal lobe to the central lobe coming to helix α3 from the other side of the α3–β6 interface. The β2–α3 loop threads through a cleft between helices α4 and α5 at the back of the central lobe. This results in the N-terminal lobe being apposed to the catalytic domain (Fig. 1A) but on the other side from the catalytic dyad (Fig. 2A). Important residues in this positioning of loop β2–α3 are Arg769, that participates in an extended network of interactions, including a salt bridge to Asp809 and a hydrogen bond to Pro808 at the base of α5; Pro777, that positions the main chain to make two hydrogen bonds to the indole ring nitrogen of Trp800 in α4; and Pro779, that inserts into a hydrophobic pocket lined by Trp800, Leu785 and Leu822. The pattern of conservation among *Tymoviridae* proteinases (Fig. 1B) indicates that this arrangement, and consequently the position of the N-terminal lobe, are very likely conserved in the family.

Remarkably, the five-residue loop between strands β5 and β6 contains two successive cis-prolines 865-Gly-Pro-Pro-867 (Fig. S3 in Text S1, Fig. 2A). Such a conformation was recently found in only 7 out of 809 Pro-Pro segments in high resolution structures of proteins [18]. Downstream of strand β6, the main chain makes a sharp turn so that the C-terminal residues of PRO 874-Lys-Arg-Leu-Leu-Gly-Ser-879 point away from the α3–β6 interface. Finally, the electrostatic potential at the surface of PRO displays three strong features (Fig. 2B): First an apolar bulge made by the two cis-prolines 866–867; second, a basic patch on the N-terminal lobe on the other side from the entry to the catalytic cleft; and third, a small acidic pocket to the side of the entry to the catalytic cleft.

### A view of the N-terminal product complex in viral polyprotein processing

**Figure 3:**
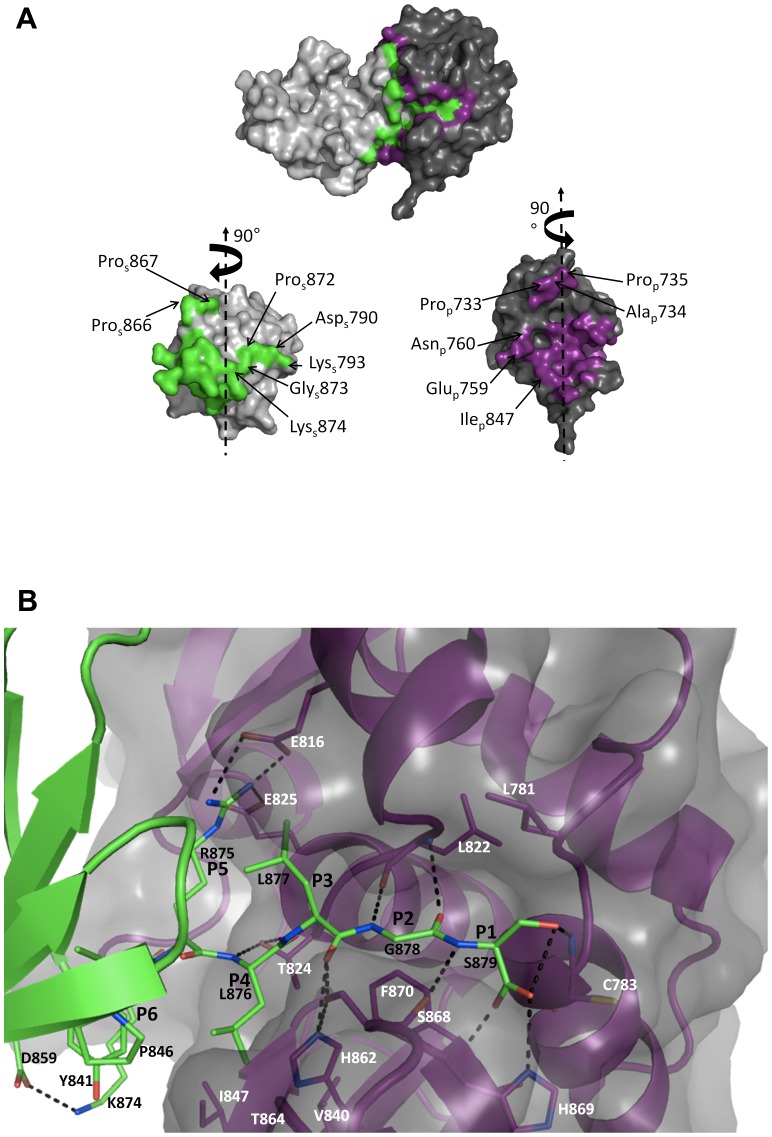
Analysis of the PRO peptidase-substrate interaction. A, Surface representation of the interface. The peptidase molecule is colored grey and substrate molecule light grey. Atoms involved in the interface are colored magenta for the peptidase and green for the substrate. Top panel: Two PRO molecules as in the crystal. Bottom panels: the two molecules have been rotated to display the surfaces of interaction. Residues in contact patches not involving recognition of the P5 to P1 substrate residues are labeled. B, Representation of interactions between P5 to P1 residues of the substrate and catalytic cleft of the peptidase. Secondary structure elements and carbons are colored magenta for the peptidase and green for the substrate. Other atoms are colored red for oxygens, blue for nitrogens and yellow for sulfur. Hydrogen bonds and salt bridges are indicated by black dotted lines. Peptidase residues are labeled in white and substrate residues in black.

The continuous helices of PRO in the crystal are formed by the insertion of the C-terminus of one molecule into the catalytic site of the next (Fig. S2 and S4 in Text S1). Thus, we have captured the N-terminal product complex resulting from the self-cleavage in *trans* of a viral polyprotein by its resident proteinase. The specificity of PRO is on the N-terminal (P) side of the scissile bond, while the C-terminal (P') side is not important as defined by mutagenesis studies [19] [8]. This structure thus reveals the molecular determinants of PRO specificity in its processing proteinase function (Fig. 3). The specificity of PRO is defined as P5-(K/R)LX(G/A/S)(G/A/S)-P1 [8]. The molecular determinants for this are now readily assigned by examining the interactions between one PRO molecule (hereafter called “substrate”, with relevant residues with an “s” subscript) and the next (hereafter called “peptidase”, with relevant residues with a “p” subscript).

Analysis of the peptidase-substrate interface using the PISA server [20] (http://www.ebi.ac.uk/msd-srv/prot_int/pistart.html) shows that 940 Å^2^ (11.9%) and 825 Å^2^ (10.5%) of solvent-accessible surface area are buried in the complex for the substrate and peptidase, respectively. This interface is not expected to be stable in solution. Accordingly, we find that PRO solutions up to 10 mg/ml are monodisperse as measured by dynamic light scattering (not shown), with an hydrodynamic radius of 2.3 nm very close to the one calculated from the crystal structure for the PRO monomer (2.14 nm). The residues involved in the interface are mostly, but not exclusively (Fig. 3A) in and around the entry to the catalytic cleft for the peptidase and in the C-terminus for the substrate, respectively. The last five residues of the substrate 875-Arg_s_-Leu_s_-Leu_s_-Gly_s_-Ser_s_-879 are funneled in an extended beta conformation towards the catalytic dyad of the peptidase Cys_p_783-His_p_869 (Fig. 3B, where the peptidase residues are labeled in white and the substrate residues in black). Indeed one of the carboxyterminal oxygens and the main chain nitrogen of Ser_s_879 are hydrogen-bonded to the main chain nitrogen and carbonyl, respectively, of Phe_p_870. Likewise, the main chain polar atoms of Gly_s_878 are hydrogen-bonded to the main chain polar atoms of Leu_p_822 in the connection between helices α6 and α7 on the other side of the catalytic cleft. The net effect is a small three-stranded intermolecular beta-sheet firmly holding 878-Gly_s_-Ser_s_-879 in place. The other Ser_s_879 carboxyterminal oxygen is hydrogen-bonded to the side chain of His_p_869. This is the only interaction stabilizing this side chain in the crystal (see above) and it is less well ordered than the other residues in the catalytic cleft (Fig. S4 in Text S1). Further upstream the substrate, the extended conformation of the main chain is maintained by hydrogen bonds from side chains of the peptidase. Key side chains are those of His_p_862 (that also participates in the P4 and P2 specificity, see below) and Thr_p_824 (Fig. 3B).

### Structural basis of PRO proteinase sequence specificity

In our crystal structure, the side chain hydroxyl of Ser_s_879 hydrogen bonds to the main chain nitrogen of the catalytic Cys_p_783 (Fig. 3B). The relaxed G/A/S specificity at P1 stems largely from the solvent exposure of the exit of the catalytic cleft (see above). Still, the side chain of Leu_p_781 caps the S1 site, precluding the presence of a large side chain at position P1. Similarly, the constriction of the active site cleft at the S2 pocket is less pronounced than in yOTU1 and vOTU, where bulky residues strictly restrict specificity to GG. The S2 pocket, lined by His_p_862, Phe_p_870 and Ser_p_868 and occupied by Gly_s_878 in our crystal structure, could readily accommodate a small side chain. At first sight, the sharp difference in specificity between P3 (seemingly no specificity) and P4 (strict specificity for a hydrophobic amino acid with a strong preference for Leu) [8] is somewhat surprising. Both Leu_s_877 (P3) and Leu_s_876 (P4) make extensive contacts to conserved, shallow hydrophobic pockets at the surface of the peptidase's central and C-terminal lobes, respectively. These contacts bury respectively 89% of Leu_s_877's and 96% of Leu_s_876's solvent-accessible surface area, as reported by PISA. Two major differences though are that Leu_s_877 (P3) is buttressed only on one side and that the S3 pocket is lined by negatively charged residues (the outer edge of the acidic pocket depicted on Fig. 2B). This explains why P3 may be mutated to Ala and may naturally be either a hydrophobic (*e.g.* Leu), small polar (*e.g.* Asn) or arginine side chain, but an aspartate is never found at this position [8]. In contrast, Leu_s_876 (P4) is sandwiched between two strictly apolar surfaces on the two C-terminal lobes of the peptidase and substrate, respectively, with His_p_862, Phe_p_870, Val_p_840, Ile_p_847 and Ser_p_842 on one side and Pro_s_846 and Tyr_s_841 on the other side. This likely accounts for the specificity at position P4. As for the strict P5 specificity for a positively charged amino acid, it is readily explained by the fact that P5 inserts its side chain into the acidic pocket depicted on Fig. 2B. Indeed, Arg_s_875 makes salt bridges to two conserved glutamates that protrude from the central lobe of the peptidase, Glu_p_816 in the small helix α6 overhanging the S3 pocket and Glu_p_825 in helix α7.

### Additional substrate recognition patches involve the peptidase's N-terminal lobe

Molecular recognition of the substrate by the peptidase further involves patches recessed from the C-terminus of the substrate (Fig. 3A). In the peptidase, these recognition patches are harbored by the N-terminal lobe. Thus, there is a prominent hydrophobic contact between the double cis-proline of the substrate Pro_s_866-Pro_s_867 and Pro_p_733-Ala_p_734-Pro_p_735 at the base of helix α1 in the N-terminal lobe of the peptidase.

A second patch in this lobe is centered on Asn_p_760 at the tip of the extended α2–β2 loop. Asn_p_760 makes both a hydrogen bond to Asp_s_790 and a stacking interaction to the Pro_s_872-Gly_s_873 motif that makes up the sharp turn to the C-terminal residues mentioned above. This contact is completed on one side by an electrostatic interaction between Glu_p_759 and Lys_s_793. On the other side, the hydrophobic contact is taken up between the aliphatic part of the side chain of Lys_s_874 and Ile_p_847, an interaction that is continuous with the S4 pocket.

### Docking of the ubiquitin/PRO complex and identification of PRO residues outside the catalytic cleft likely involved in ubiquitin recognition

**Figure 4:**
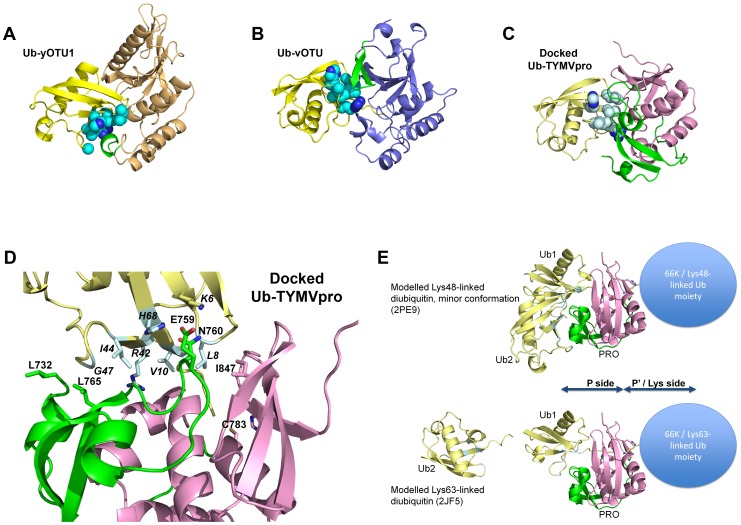
Docking of the TYMV DUB/ubiquitin complex. In all panels, molecules are displayed as ribbons and ubiquitin is colored yellow with its Ile44 patch in cyan (spheres in panels *A*, *B*, *C*, sticks in panel *D*). The ubiquitin colors are brighter in the actual crystal structures of complexes (Ub-yOTU1 and Ub-vOTU). yOTU1, vOTU and TYMVpro are in the same orientation after superposition by DALI in panels *A*, *B* and *C*. A, Ub-yOTU1 complex crystal structure. yOTU1 is in light orange, with a key element contacting the Ile44 patch in green. B, Ub-vOTU complex crystal structure. vOTU is in light blue and as in A, a key vOTU element contacting the Ile44 patch is in green. C, Ub-TYMVpro docked model. The PRO catalytic domain is in pink and its N-terminal lobe in green. D, Close up of the predicted interaction between TYMVpro and Ubiquitin. The catalytic cysteine is displayed as sticks and labeled, as well as PRO residues that were mutated for our deubiquitylation assay. Ubiquitin residues putatively contacted by PRO are labeled in italics. E, Further modeling of diubiquitin/PRO complexes. PRO is colored as in C and labeled. The two ubiquitin moieties are labeled, with Ub1 the moiety that has been superposed to the ubiquitin in the docked model of CD. The approximate location of the ubiquitylated substrate is depicted as a blue balloon with a lysine side chain pointing into the active site. Top, Lys48-linked diubiquitin, in the minor conformation detected by nuclear magnetic resonance. Bottom, Lys63-linked diubiquitin, in the extended conformation seen in crystal structures.

Using the HADDOCK program (see Materials and Methods), we performed a docking simulation to explore the possible binding modes of ubiquitin in association with PRO. We first defined spatial ambiguous restraints for the interaction between ubiquitin and PRO by the involvement of: 1) the C-terminal residues of Ub 2) the corresponding catalytic cleft on PRO and 3) an apolar patch on the surface of ubiquitin (hereafter referred to as the Ile44 patch) that is recognized by most ubiquitin-binding proteins [21]. The ubiquitin residues most frequently targeted in this patch are Ile44, His68, Val70, Gly47, Leu8 and Arg42 (in cyan on Fig. 4) [21]. The structures of both yOTU1 and vOTU have been reported as covalent complexes with ubiquitin [13] [14] [15] [16]. These works have shown [14] [15] that the Ile44 patch is recognized by nonhomologous regions of the two OTU DUBs due to a 75° rotation of Ub around an axis defined by the main chain of the 5 C-terminal Ub residues (Fig. 4AB). To account for this known flexibility of the C-terminal tail of Ub [21], multiple conformations were sampled prior to the rigid-body docking step so as not to bias the interface too heavily towards a given binding mode. Two clusters of solutions were found, among which only the largest cluster (which also contains the complex with the best HADDOCK score) had a binding mode consistent with the C-terminal residues of Ub in the PRO active site. This binding mode was cross-confirmed by other docking simulations using alternative methodologies, in particular without applying prior restraints between the putative binding regions (see Protocol S2 in Text S1). Further inspection of the lowest energy structure (Fig. 4C) shows that the orientation of ubiquitin is similar to that in the vOTU complex and that the predicted interface prominently involves PRO's N-terminal lobe. Indeed, the tip of the lobe's extended α2–β2 loop inserts into the patch (Fig. 4D), suggesting that Glu759 and Asn760, that participate in PRO_s_ binding (Fig. 3A), may also be involved in ubiquitin recognition. Indeed, in the docking model Glu759 would be in a position to make salt bridges to Ub His68 in the Ile patch and/or Lys6 at its periphery. Since such interactions are usually good specificity but weak affinity determinants, we further looked for hydrophobic patches on PRO's surface that could come into contact with the Ile44 patch based on the model prediction. We found two such PRO patches on either side of the Ile44 patch (Fig. 4D). One is made by Leu732/Leu765 in the N-terminal lobe. The other is centered on Ile847. Although not part of the N-terminal lobe, Ile847 seemed an excellent candidate as it is an exposed residue with a conserved hydrophobic character (Fig. 1B). This matches a known feature of interface evolution, where contacts between apolar patches are the most conserved although the residues themselves may not be [22]. Thus if the docking model was correct and Ile847 was an interface residue with the Ile44 patch, we expected a reduction in the bulk of its side chain to reduce the interaction and the substitution for a short charged residue to almost abolish it.

### Probing the contributions of residues in and around the N-terminal lobe to the DUB activity of PRO

In view of the docking results, we assessed the DUB activity of PRO and selected mutants. All mutants described below were produced in *E. coli* in a soluble form and purified to homogeneity (Fig. S6 in Text S1). Dynamic light scattering analysis of the mutants showed the same results as for the wild type, indicating that they were likely properly folded.

**Figure 5:**
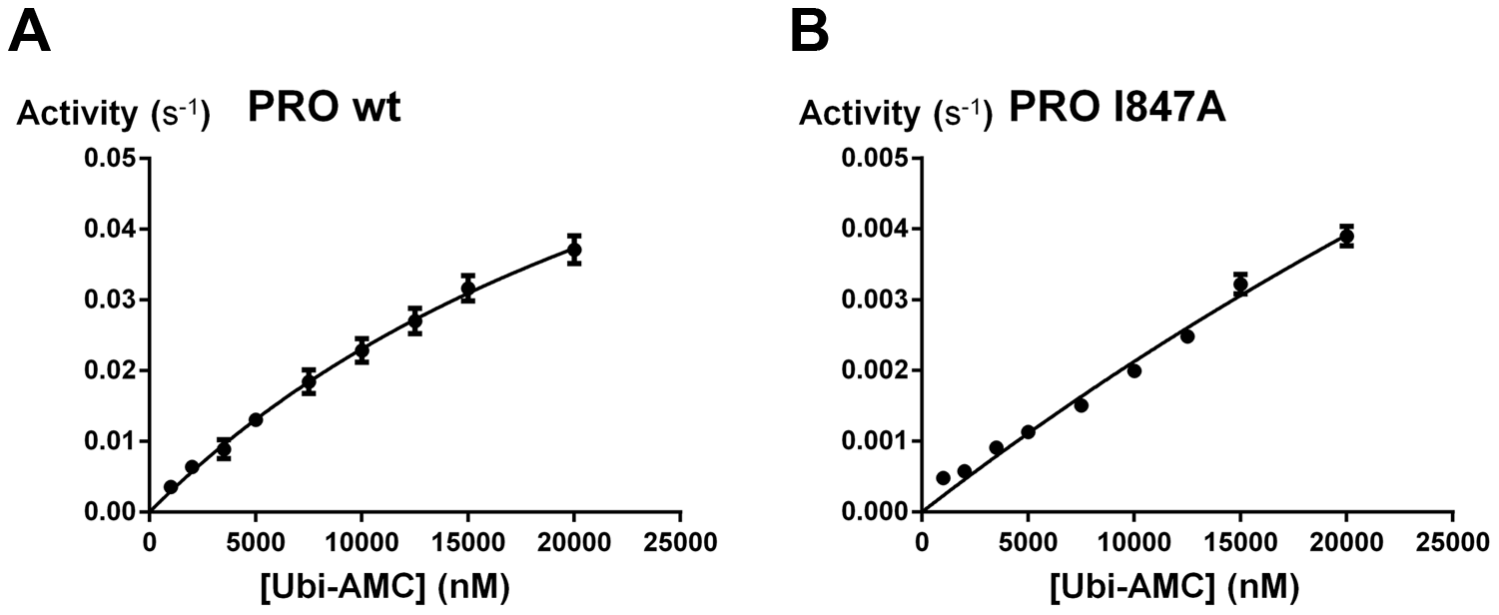
DUB activity of the wild type PRO and I847A mutant. **Initial velocities of AMC release as a function of Ub-AMC concentration.** For easier comparison, values are expressed as amount of product released per second per amount of enzyme. A, Wild type PRO. B, I847A mutant.

As a deubiquitylating assay we used hydrolysis of the general substrate Ubiquitin-7-amino-4-methylcoumarin (Ub-AMC). Determination of initial velocities up to the highest Ub-AMC concentration available to us showed that the wild type hexahistidine-tagged PRO whose structure is reported here is still far from saturating conditions at the highest substrate concentration we could reach (20 µM Ub-AMC, Fig. 5A). Accordingly, we compared the wild type and mutant enzymes by determining their pseudo first-order rate constants, Kapp, which approximate kcat/Km in conditions far from saturation (Table 2). For wild type PRO, the K_app_ value of 2650 M^−1^s^−1^ we find is comparable to the K_app_ of 1550 M^−1^s^−1^ previously reported from initial velocity measurements of a GST-tagged version of PRO [9] and thus ∼100-fold less than the K_app_ reported for vOTU [14] [15] [16].

**Table 2:** K_app_ constants of PRO and selected mutants in an Ub-AMC hydrolysis assay far from saturating conditions.

	K_app_ (M^−1^s^−1^)*
PROwt	2648±123
L732A/L765A	2507±196
E759G/N760G	2062±196**
I847A	215±5
I847D	14±1

* K_app_ is reported according to the equation V/[E] = kapp [S]. All experiments were performed three to five times. Data are expressed as the averages and standard deviations of these independent experiments.

** significantly lower than PROwt (p<0.01) in the Mann-Whitney rank test using the five experiments for each enzyme.

A L732A/L765A mutant was not significantly affected in its K_app_ (p = 0.34, Mann-Whitney rank test). On the other hand, an E759G/N760G mutant showed a significant (p<0.01) though slight (20%) reduction in K_app_, suggesting that the α2–β2 loop is indeed involved in ubiquitin recognition. Further tampering with this loop, *e.g.* deleting its tip by replacing 758-PENT-761 with a diglycine motif, led to no soluble PRO production, so that we could not further probe this.

We next assessed Ile847 mutants for their DUB activity. I847A is impaired in K_app_ (a 10-fold deterioration), while I847D is barely active (a 150-fold reduction in K_app_). This behavior is exactly as predicted from the docking model, since I847A will reduce the size and complementarity of the PRO hydrophobic patch, while I847D will destroy its apolar character altogether. To further probe the docking model, we tested the I847A mutant initial velocity at higher substrate conditions (Fig. 5B), where the slope of the wild type curve starts to decrease (Fig. 5A). Within the same range, the I847A curve still appears linear in substrate concentration, suggesting that ubiquitin binding rather than turnover rate is impaired in the I847 mutants.

## Discussion

### A view of the TYMV 206K polyprotein cleavage in *trans*

In the present work, we provide structural insights into viral polyprotein processing by a viral proteinase that cleaves at its own C-terminus. Such an event is common enough in the viral world, particularly among positive-stranded RNA viruses, and may in principle be achieved either in *cis* (the proteinase domain cleaves the polypeptide of which it is a part) or in *trans* (it cleaves another polyprotein molecule). Our structure precludes the possibility of the C-terminus of PRO looping back towards the entry to the catalytic cleft in the same molecule. Therefore, cleavage of the TYMV replication polyprotein at the PRO/HEL junction occurs only in *trans*. This cleavage is a regulatory event in the replication of the TYMV RNA genome. It occurs in the replication complex comprising the two products of the first cleavage: the 66K RdRp and the 140K protein. 140K harbors PRO and localization determinants to the chloroplast envelope, where it recruits 66K. There, cleavage of 140K at the PRO/HEL junction into 98K and the 42K helicase contributes to the switch to synthesis of the +strand [7] [8] (Fig. S1 in Text S1). A strictly *trans* cleavage likely takes part in the regulation by requiring a sufficient local concentration of 140K at the chloroplast membrane and/or remodeling of this membrane into a special compartment for viral replication before synthesis of new viral genomes.

### A proteinase with an exposed active site allowing relaxed specificity

The interface in the crystal of the N-terminal complex of this *trans* cleavage reveals the molecular determinants for the peptidase sequence specificity. The fact that PRO proteinase specificity is confined to the P side is readily explained by the fact that the catalytic cleft ends abruptly at the catalytic dyad, leaving it completely solvent-exposed. Thus, residues on the P' side of the substrates will have little or no contact with PRO. In contrast, there are extensive interactions with the P-side residues up to P5 from both lobes making up the catalytic proteinase domain. The C-terminal β-sheet lobe thus provides a hydrophobic pocket for P4 and the central α-helical lobe an acidic pocket contributing salt bridges to the positively charged P5 and a shallow hydrophobic patch for P3. The constriction of the active site cleft at the interface between the two lobes ensures that only small residues (but not necessarily glycines) can be at positions P2 and P1.

We recently reported that 98K has DUB activity *in vivo* and *in vitro* and that this DUB activity is localized in PRO [9]. Thus, PRO recognizes at least three different substrates that differ at positions P1 (G for Ub, but A and S for the HEL/66K and PRO/HEL junctions, respectively) and specifically cleaves either endopeptide bonds (HEL/66K, PRO/HEL) or isopeptide bonds (Ub). Our crystal structure shows that the latter property is linked to an unusual solvent exposure of the active site of PRO on the P'/lysine sidechain side of the cleavage. The former property of relaxed sequence specificity is allowed by an also unusual lesser constriction of the PRO active site cleft at the P1 and P2 positions (see below). Such features imply that PRO may be more heavily dependent on the recognition of additional molecular determinants away from the active site, in order to maintain sufficient substrate affinity and most importantly, high substrate specificity.

### A distal target recognition lobe and a minimal active site

Accordingly, the crystal structure we obtained allows us to identify two such determinants. First, an acidic pocket to the side of the entry to the active site strongly favors a positively charged residue in P5 of the substrate. Second and most important, we identify the *Tymoviridae*-specific N-terminal lobe of PRO as a recognition element for surface patches of the PRO/HEL substrate, as this lobe was found to recognize a signature bulge made of the two successive cis-prolines in the PRO substrate molecule. Whether the N-terminal lobe is also prominently used in recognition of the HEL/66K junction cannot be assessed at present. However, docking of the PRO/Ub complex and subsequent mutational analysis of the DUB activity of PRO suggest that PRO targets the Ile44 patch that is recognized by all characterized DUBs in part with elements also involved in recognition of PRO_s_, such as Ile847 and possibly the α2–β2 loop. Of note, our docking model places the three residues that differ between plant ubiquitin (the natural TYMV PRO target) and human ubiquitin (that we used in modeling and functional work) on the side of ubiquitin opposite the interfaces with PRO (Fig. S7 in Text S1). This would rule out a different behavior of the natural substrate of PRO's DUB function (plant ubiquitin) compared to the readily available experimental substrates (derivatives of human ubiquitin).

Using a myc-tagged version of human ubiquitin, it was previously shown that, in contrast to other viral DUBs (*e.g.* vOTU), 98K is a very specific DUB whose overexpression in cells does not lead to a global deubiquitylation of cellular proteins but rather to specific deubiquitylation of 66K [9]. Several lysine side chains of 66K are polyubiquitylated *in vivo* [10] and the types of these ubiquitin chain linkages are presently unknown. *In vitro* TYMV PRO may disassemble both Lys48-linked and Lys63-linked polyubiquitin chains, albeit with weak activity [9]. In the light of our findings, one may ask whether PRO may display specificity to particular ubiquitin linkages. Specificity may be achieved in several ways, *e.g.* on the P'/Lys side of the catalytic cleft (Fig. 4E) either by recognizing the sequence context of the modified lysine or by positioning the Lys-linked moiety. In either case, addressing the question of specificity would require modeling a diubiquitin chain across PRO's catalytic site. Such an exercise (not shown) must be highly speculative at the moment in the absence of structures for relevant complexes of OTU DUBs [23]. We may note that, as for other OTU DUBs, the isopeptide bonds in extended linkages (such as Lys63-linked polyubiquitin) can in principle be readily accessed by PRO, but compact chain conformations (as in Lys48-linked polyubiquitin) require an extensive conformational change to expose the isopeptide bond and allow binding and cleavage by PRO [23]. But the question of linkage specificity can also be addressed by modeling a diubiquitin chain on the P side of PRO's catalytic cleft (Fig. 4E). Molecular recognition of Lys48-linked chains is poorly understood, as in their compact conformations their ubiquitin moieties interact through their Ile44 patches. It is proposed that structural flexibility allows transient access to the Ile44 patches to binding partners, and indeed a minor population of more open Lys48-linked diubiquitin has been modeled from nuclear magnetic resonance data [23]. Interestingly, placing this minor conformation onto our docking model results in PRO's N-terminal lobe being sandwiched between the catalytic domain and the Lys48-linked diubiquitin and making contact with both ot the latter's Ile44 patches (Fig. 4E, top). On the other hand, similarly placing the structure of a Lys63-linked diubiquitin predicts no interactions to the second moiety (Fig. 4E, bottom), due to the extended character of the Lys63 linkage.

PRO counteracts the 66K polymerase degradation by the ubiquitin-proteasome system through polyubiquitin removal [10] [9]. Since Lys48-linked polyubiquitylation is the canonical proteasome addressing signal, simultaneous recognition by the N-terminal lobe of several ubiquitin moieties on the P side (Fig. 4E, top) could be a mechanism allowing more efficient cleavage of Lys48-linked polyubiquitin chains. It might also explain in part why PRO displays rather poor activity for a DUB (*e.g.* compared to vOTU [14] [15] [16]) in a general deubiquitylation assay using a monoubiquitin derivative [9] (this work), with a Km in the tens of micromolar range (Fig. 5A). The other obvious feature of TYMV PRO explaining its lesser activity is the minimal character of its active site. It lacks altogether two important functional elements that are present in most cysteine proteinases, including the closest relatives of PRO (clan CA, including yOTU1 and vOTU, see below): The oxyanion hole and a general acid as the third catalytic residue. Our structural work thus draws the picture of a barely complete proteinase that nonetheless effectively achieves cleavage of several endo- and isopeptide targets by combining co-localization with the targets and a versatile recognition lobe.

### A peculiar proteinase domain

Among peptidases that process polyproteins from RNA viruses with a Cys/His catalytic dyad, there are two known structural clans with unrelated folds. The first is clan CA, that comprises yOTU1 and vOTU. Another is clan CN, whose type is the nsP2 proteinase of alphaviruses [24]. Alphaviruses, including Sindbis virus, Semliki Forest Virus and Chikungunya virus, are animal relatives of tymoviruses. The two virus families share many features in their replication strategies, including successive cleavages of the replication polyprotein by the resident proteinase regulating RNA+ vs −strand synthesis [8]. Nevertheless, our data clearly show that PRO is unrelated to nsP2 and assign PRO to clan CA, a result that could not be firmly established by sequence comparisons alone (http://merops.sanger.ac.uk/) [25] [9]. The two other families of processing proteinases assigned to clan CA are also from positive-stranded RNA viruses: They are the coronavirus papain-like proteinases PLP1 and PLP2 [26] [27] and the picornavirus leader proteinase [28]. These proteinases have also been reported to be ubiquitin hydrolases [27] [29]. Yet PRO does not display detectable homology to these proteinases.

Instead, the fold of PRO's two-lobed catalytic domain is clearly a more compact version of the OTU domain fold of ubiquitin hydrolases. The least dissimilar OTU domains to PRO are those of the cellular OTU1 DUB (yOTU1), whose structure is available in complex with Ub [13], and the viral OTU domain encoded in the L protein of Crimean–Congo haemorrhagic fever virus (vOTU), whose structure has been recently reported in complex with Ub and ISG15 [14] [15] [16]. Thus, PRO is closest to enzymes with no endopeptidase activity.

Potential clues as to how TYMV acquired an ubiquitin hydrolase as a dual DUB/processing proteinase may be found in the family *Flexiviridae* of plant viruses. In this closest family to *Tymoviridae*, some of the replication proteins encode two peptidase domains, an OTU domain being N-terminal to the processing proteinase P [30]. One may therefore picture a scenario in which an ancestor to *Tymoviridae* harbored such a two-peptidase replication polyprotein. Subsequently, the OTU peptidase acquired specificity determinants allowing its use as processing proteinase and the P domain was lost. This report and previous works [13] [14] [15] establish that nonhomologous recognition modules have repeatedly evolved in the OTU family of DUBs, which is consistent with such a scenario. Whatever actually happened, the present diversity of specific functions performed by PRO is remarkable in a proteinase domain that is no larger (148 ordered residues) than the more specialized vOTU (162 ordered residues) or yOTU1 (170 ordered residues). Our results shed light on the molecular details that allow such a compact protein to perform a diversity of key functions in viral genome replication and host-pathogen interaction.

## Materials and Methods

### Protein expression, purification, and crystallization

The production and purification of an N-terminally 6-histidine tagged PRO domain and of PRO mutants are described in details in protocol S1 in Text S1. Briefly, the coding sequence of the PRO domain (residues 728–879 of 206K) was produced with an in-frame N-terminal 6His-tag. Purification was performed with two successive chromatography steps (immobilized metal affinity chromatography followed by size exclusion chromatography). Crystallization is described elsewhere [11]. Briefly, a pool from all fractions of the size exclusion step in buffer 10 mM Tris-HCl pH 8, 350 mM Ammonium Acetate, 1 mM DTT, was concentrated to 39 mg/ml as judged by OD280 nm. Hexagonal crystals of up to 50×50×40 µm^3^ grew in a single vapor diffusion drop where 1 µl protein solution plus 1 µl well solution (0.1 M Hepes pH 7.5, 2.5 M Ammonium formate) was equilibrated against a 0.5 ml reservoir volume. Prior to testing, crystals were transferred for ∼30 s in 0.1 M Hepes pH 7.5, 4 M Ammonium formate, 16% glycerol and flash cooled by plunging into liquid nitrogen.

### MIRAS phasing, model building and refinement

Details of the structure determination are given elsewhere [11]. Briefly, the structure was solved by MIRAS from three poor derivatives thanks to the high (69%) solvent content of the crystals. Heavy atom derivatives (HgAc_2_, NaI and CsCl) were obtained by soaking. Data were processed with the XDS package [31]. Initial heavy atom sites were located with SHELXD [32]. This first heavy atom model was refined, completed and pruned and initial phases were computed and improved with autoSHARP [33]. The resulting map was interpretable and a first model was built with phenix.autobuild [34]. The model was manually rebuilt with COOT [35] and refined with phenix.refine [34]. Data processing and refinement statistics are collated in table 1.

### Structure analysis

Interfaces in the crystal were assessed using the PISA server [20] (http://www.ebi.ac.uk/msd-srv/prot_int/pistart.html). Homologs of PRO were sought and superimposed with the DALI server [12] (http://ekhidna.biocenter.helsinki.fi/dali_server). Structures were displayed and figures were prepared with Pymol (www.pymol.org). Figure 1B was generated with *ALINE* [36].

### Docking of ubiquitin onto the PRO structure and subsequent diubiquitin modeling

98 monomeric structures were generated from the Ub monomer extracted from the vOTU-Ub structure by sampling and clustering 2,000 C-terminal tail conformations using the Rosetta 3.4 FloppyTail application [37]. These conformations were used as a starting ensemble for Ub in the docking process. HADDOCK v2.1 [38] [39] was used to perform the docking with standard parameters, generating 5,000 rigid-body docking conformations followed by flexible explicit solvent refinement of the best 500 structures. The solutions were clustered and the most likely model was picked (see details in Protocol S2 and Fig. S5 in Text S1).

This model was subsequently used for visualizing PRO/diubiquitin models. Lys48-linked diubiquitin was either the compact structure (PDB 1AAR) or the minor population structure (PDB 2PE9) [23]. Lys63-linked diubiquitin was PDB 2JF5. For each diubiquitin, one moiety was superimposed on the ubiquitin in the docking model. This was either the moiety with the lysine-linked C-terminus (across-cleft modeling) or the moiety with the free C-terminus (P side modeling). In across-cleft modeling, this results in major clashes of the other moiety with PRO for both Lys48-linked diubiquitin conformations and still large clashes for Lys63-linked diubiquitin. In P side modeling, this results also in unrelievable clashes for the compact Lys48-linked diubiquitin, as with all proteins binding the ubiquitin Ile44 patch. There were few clashes with the minor population Lys48-linked diubiquitin in P side modeling and none with the Lys63-linked diubiquitin.

### Deubiquitylation assay

Recombinant wild type and mutant his-PRO were generated, produced and purified as described in Protocol S1 in Text S1. Samples were concentrated to 200–1096 µM, dialyzed in 50 mM HEPES pH 8, 150 mM KCl, 1 mM DTT, 10% glycerol, aliquoted and kept at −80°C until use. DUB activity was assessed in Assay buffer (HEPES-KOH 50 mM pH 7.8, KCl 10 mM, EDTA 0.5 mM, DTT 5 mM, NP40 0.5%, DMSO 2%) using the fluorogenic substrate Ub-AMC (Boston Biochem). DMSO was adjusted to 2% in all assays to match the DMSO concentration in the highest Ub-AMC concentration tests. The rate of substrate hydrolysis was determined by monitoring AMC-released fluorescence as described previously [9] with some modifications. Assays were performed at 20°C in a temperature-controlled Perkin-Elmer LS50B spectrofluorimeter. The initial velocity V was derived from the linear increase in fluorescence at 460 nm (excitation at 380 nm) in minutes 1 to 11 after mixing in Ub-AMC.

In order to determine the K_app_, the substrate concentration was kept at a concentration below 0.5 µM where the initial velocity is linear in substrate concentration. Enzyme concentrations were 100 nM for wild type PRO, L732A/L765A and E759G/N760G, 1 µM for I847A and I847D. The apparent k_cat_/K_m_ (K_app_) values were determined according to the equation V/[E] = Kapp/[S].

Subsequently V was also determined at higher substrate concentrations ranging from 1 µM to 20 µM for PRO wild type (10 nM) and Pro I847A (100 nM). Results were fitted to Michaelis-Menten kinetics by nonlinear curve fitting using Graphpad Prism (Graphpad Software inc., la Jolla, CA).

Data were expressed as the means and standard deviations of these independent experiments. All experiments were performed at least in triplicates for K_app_ values and at least in duplicates for the higher substrate concentrations experiments.

## Supporting Information

Text S1 **Supplementary protocols and figures.** Protocols S1–S2 and figures S1–S7.
(PDF)
