# Supplementary material for: A Compact Viral Processing Proteinase/Ubiquitin Hydrolase from the OTU Family
Source: PLoS Pathog. 2013 Aug 15;9(8):e1003560. doi: 10.1371/journal.ppat.1003560 (PMC3744425; doi:10.1371/journal.ppat.1003560)
Supplement: Text S1 — Supplementary protocols and figures. Protocols S1–S2 and figures S1–S7. (PDF) [file ppat.1003560.s001.pdf]

Supporting information

Protocol S1

Expression, purification, and mutagenesis of TYMV his-PRO

An N-terminally 6-histidine tagged PRO domain was obtained as follows: We constructed the bacterial expression vector pHis-PRO encoding TYMV PRO domain (residues 728-879 of TYMV 206K protein, Uniprot accession P10358) fused to an in-frame N-terminal hexahistidine tag (the complete sequence encoded is given in Fig. 1B). All DNA manipulations were performed using standard techniques. pHis-PRO was obtained from pGex-PRO [1] as follows: a unique *Nde*I restriction site was first created upstream of the GST ORF by site directed mutagenesis using primers 5'-ACAGGAAACAGTACATATGTCCCCTATCCTAGGTTATTGGAAAATTAAG-3' and 5'-TCCAATAACCTAGGATAGGGGACATATGTACTGTTTCCTGTGTGAAATTGTTATCCGCTCACAATTCCACACATTATACGAGCCGA-3'. The *Nde*I-*Bam*HI fragment corresponding to the GST ORF was then replaced by the hybridized complementary adaptors: 5'-TATGCACCATCACCATCACCATGGTAC-3' and 5'-CATGGTGATGGTGATGGTGCA-3'.

Site-directed mutagenesis to obtain mutant pHis-PRO was performed using the QuikChange® II Site-Directed Mutagenesis Kit (Agilent), or by custom services of ShineGene Bio-Technologies, Inc. All clones were verified by DNA sequencing.

Expression plasmids were transformed into *Escherichia coli* BL21 rosetta (DE3). For each construct, n overnight culture was used to inoculate 1-L of LB media containing 50 µg/L carbenicillin and 25 µg/L chloramphenicol. This culture was grown at 37°C to optical density (OD600) 0.6. Expression was induced by the addition of 0.5 mM isopropyl-b-D-thiogalactopyranoside (IPTG) and the cells were grown for 4 hours at 30°C. The cell pellet was harvested, frozen and stored at -20°C.

The pellet was resuspended in 20 mL of lysis buffer (100 mM Tris-HCl pH 7.5, 350 mM NaCl, 25 mM imidazole, 1 mM DTT, 0.5 % Triton X-100, 2 mg/ml Lysozyme, and 1 U/ml Benzonase) and incubated for 60 min on ice. Lysis was completed by 5 freeze/thaw cycles (70 K/303 K).

The disrupted cell lysate was centrifuged at 8000 xg during 30 min and the supernatant was loaded onto a 1 mL Ni2+-NTA agarose column (Qiagen) preequilibrated with buffer A (100 mM Tris-HCl pH 7.5, 350 mM NaCl, 25 mM imidazole, 1 mM DTT). The column was washed with 50 mL of buffer A, followed by 10 mL of washing buffer A^2 (100 mM Tris-HCl, pH 6.0, 350 mM NaCl, 25 mM imidazole, 1 mM DTT). The protein was eluted by the elution buffer B (100 mM Tris-Cl, pH 7.5, 350 mM NaCl, 500 mM imidazole, 1 mM DTT). The eluted PRO was further purified by high-resolution Superdex S-75 gel filtration column (Amersham) with Buffer C (10 mM Tris-HCl pH8, 350 mM Ammonium Acetate, 1mM DTT). For DUB activity assays, we pooled only those fractions that were not contaminated by

the bacterial S15, yielding electrophoretically pure PRO as judged by Coomassie-stained SDS-PAGE (Fig. S6).

Crystallization.

A pool from all fractions of the gel filtration step in buffer C was concentrated to 39 mg/ml as judged by OD280 nm. This sample was thus contaminated with bacterial protein S15 [2]. Showers of needles and hexagonal crystals of up to 50 x 50 x 40 μm^3 grew in a single vapor diffusion drop where 1 µl protein solution plus 1 µl well solution (0.1M Hepes pH 7.5, 2.5 M Ammonium formate) was equilibrated against a 0.5 ml reservoir volume. Similar needles also appeared with pure PRO samples, but the hexagons were obtained only from this contaminated preparation and turned out to contain one PRO and one S15 molecule par asymmetric unit [2]. Prior to testing, crystals were transferred for ~30 s in 0.1M Hepes pH 7.5, 4 M Ammonium formate, 16% glycerol and flash frozen by plunging into liquid nitrogen. The hexagons diffracted to close to 2 Å resolution on synchrotron beamlines. We note that S15 is a much better Coomassie binder than PRO, comprising 12.4% or Arg residues (4.4% in PRO) [3]. The S15 contamination was thus far from stoechiometric and amounted to a few percent in mass (see Fig.1 in [2]), possibly some 10% mol/mol. Since on the other hand, S15 is less well detected at OD280nm (calculated absorbance of 0.29 cm^{-1} $(mg/ml)^{-1}$) than PRO (calculated absorbance of 0.54 cm^{-1} $(mg/ml)^{-1}$), the actual PRO concentration was close to that calculated by OD280 nm without taking S15 into account.

Protocol S2

Docking of ubiquitin onto the PRO structure.

Detailed procedure.

2,000 monomeric structures were generated starting from the Ub monomer extracted from the vOTU/Ub structure, maintaining the structure for residues 1-70 and sampling C-terminal tail conformations using the Rosetta 3.4 FloppyTail application [4] with standard parameters. These conformations were clustered at 0.2 Å backbone RMSD. For each of the resulting 98 clusters, a representative conformation was picked.

These 98 conformations were used as a starting ensemble for Ub in the docking process. The docking software HADDOCK v2.1 [5] [6] was used with standard parameters. The ambiguous restraints for the HADDOCK run were defined from examining the structures of vOTU/Ub and cOTU/Ub: the active residues were selected as L822, S823, T824, E825 from the groove on PRO, and L8, I44, V70, R72, L73, R74 on Ub. The passive residues were defined as all solvent-accessible neighbors of the active residues, namely positions 760, 763, 764, 765, 811, 816, 820, 821, 822, 823, 824, 825, 847, 849, 862, 868 on PRO and positions 4, 6, 7, 9, 10, 11, 12, 27, 26, 28, 29, 40, 41, 42, 43, 45, 46, 47, 48, 49, 66, 68, 69, 71, 72, 73, 74 on Ub. Initially 5,000 rigid-body docking conformations were generated. Of these, the best 500 structures were subsequently refined allowing the sidechains to be flexible. A final

refinement in explicit solvent was performed. The 500 structures thus generated were then clustered on the basis of their mutual interface backbone RMSDs and their HADDOCK scores (see Fig. S5).

Other docking simulations.

Other docking simulations were performed in order to test the robustness of the binding mode obtained with the HADDOCK method. In particular, we ran docking simulations without applying any prior restraints between the putative binding regions.

First, 54,000 rigid-body conformations for the PRO/Ub interface were generated using Zdock [7]. Zrank [8] was used to rerank those structures but no likely cluster of solutions emerged. Scoring these decoys using InterEvScore, a novel scoring function combining a multi-body statistical potential with evolutionary information [9], led to identification of a well-ranked solution with 1.73 Å interface backbone RMSD to the model chosen from the HADDOCK simulations above.

We also tested the extent to which the extended shape of the C-terminal tail of Ub constrained the docking solutions. A shortened version of Ub lacking the C-terminal flexible tail (Ub1-71) was docked onto PRO using HADDOCK with a set of ambiguous restraints between the active residues (E825, I847 and F849 on PRO; L8, I44 and V70 on Ub) and the passive residues (positions 758, 759, 760, 761, 763, 764, 781, 782, 783, 784, 816, 817, 819, 820, 821, 822, 823, 824, 840, 842, 843, 844, 845, 846, 848, 862, 864, 868, 869, 870, 877 on PRO and positions 4, 6, 7, 9, 10, 11, 12, 27, 26, 28, 29, 40, 41, 42, 43, 45, 46, 47, 48, 49, 66, 68, 69, 71 on Ub). Generation of 1,000 conformations through rigid-body docking was followed by refinement of the 200 best structures, which were then clustered. The largest cluster had an orientation of Ub precluding the insertion of the C-terminal tail in PRO’s catalytic cleft. In contrast, the second largest cluster is compatible with such an insertion. The interface with the best HADDOCK score belongs to this second largest cluster and has a low interface backbone RMSD of 2.02 Å compared to the model chosen in the HADDOCK run with full-length Ub. This interface also involves interactions between the N-terminal lobe and Ile847 on PRO and the apolar patch on Ub supporting the selection of the model presented in the main text. Taken together, these results indicate a common most likely binding mode for the PRO/Ub interface, which was further confirmed by directed site mutagenesis experiments on Ile847, E759/N760 and L732/L765.

References

1. Chenon M, Camborde L, Cheminant S, Jupin I (2012) A viral deubiquitylating enzyme targets viral RNA-dependent RNA polymerase and affects viral infectivity. EMBO J 31: 741–753. doi:10.1038/emboj.2011.424.

2. Robin C, Beaurepaire L, Chenon M, Jupin I, Bressanelli S (2012) In praise of impurity: 30S ribosomal S15 protein-assisted crystallization of turnip yellow mosaic virus proteinase. Acta Crystallogr Sect F Struct Biol Cryst Commun 68: 486–490. doi:10.1107/S1744309112008445.

3. De Moreno MR, Smith JF, Smith RV (1986) Mechanism studies of coomassie blue and silver staining of proteins. J Pharm Sci 75: 907–911.

4. Kleiger G, Saha A, Lewis S, Kuhlman B, Deshaies RJ (2009) Rapid E2-E3 assembly and disassembly enable processive ubiquitylation of cullin-RING ubiquitin ligase substrates. Cell 139: 957–968. doi:10.1016/j.cell.2009.10.030.

5. Dominguez C, Boelens R, Bonvin AMJJ (2003) HADDOCK: a protein-protein docking approach based on biochemical or biophysical information. J Am Chem Soc 125: 1731–1737. doi:10.1021/ja026939x.

6. De Vries SJ, van Dijk ADJ, Krzeminski M, van Dijk M, Thureau A, et al. (2007) HADDOCK versus HADDOCK: new features and performance of HADDOCK2.0 on the CAPRI targets. Proteins 69: 726–733. doi:10.1002/prot.21723.

7. Chen R, Li L, Weng Z (2003) ZDOCK: an initial-stage protein-docking algorithm. Proteins 52: 80–87. doi:10.1002/prot.10389.

8. Pierce B, Weng Z (2007) ZRANK: reranking protein docking predictions with an optimized energy function. Proteins 67: 1078–1086. doi:10.1002/prot.21373.

9. Andreani J, Faure G, Guerois R (2013) InterEvScore: a novel coarse-grained interface scoring function using a multi-body statistical potential coupled to evolution. Bioinformatics. doi:10.1093/bioinformatics/btt260.

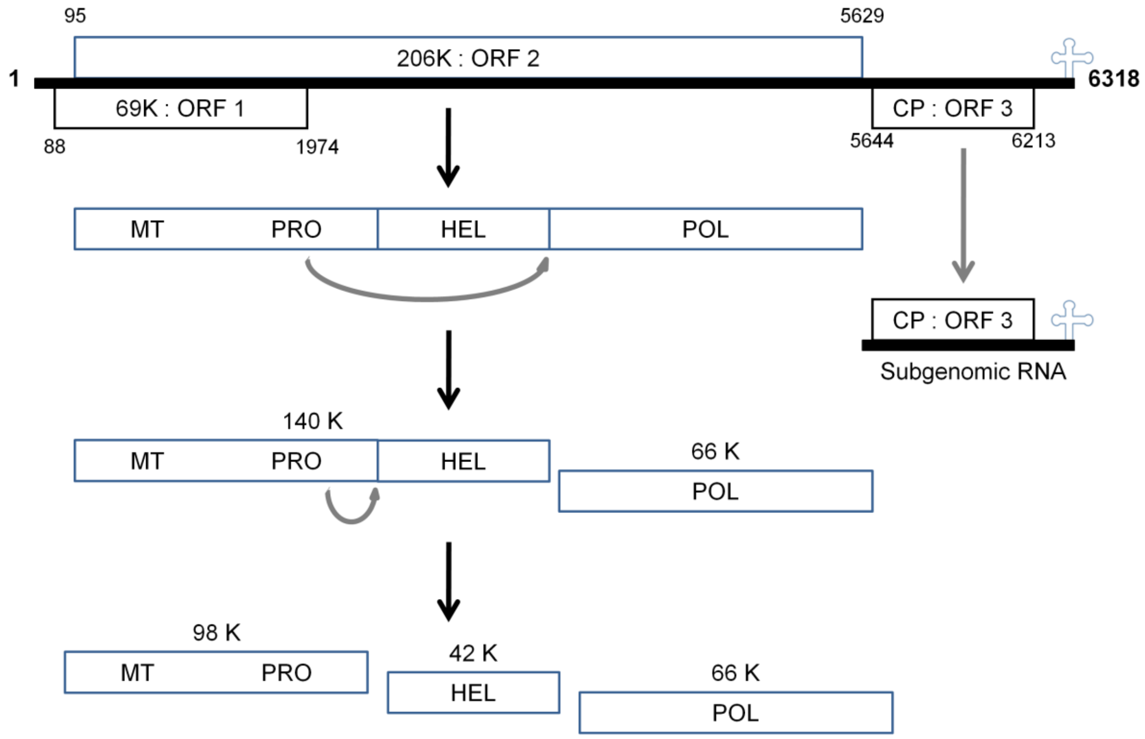

Figure S1

Figure S1: Genomic organization of TYMV RNA and proteolytic processing by PRO of the 206K polyprotein.

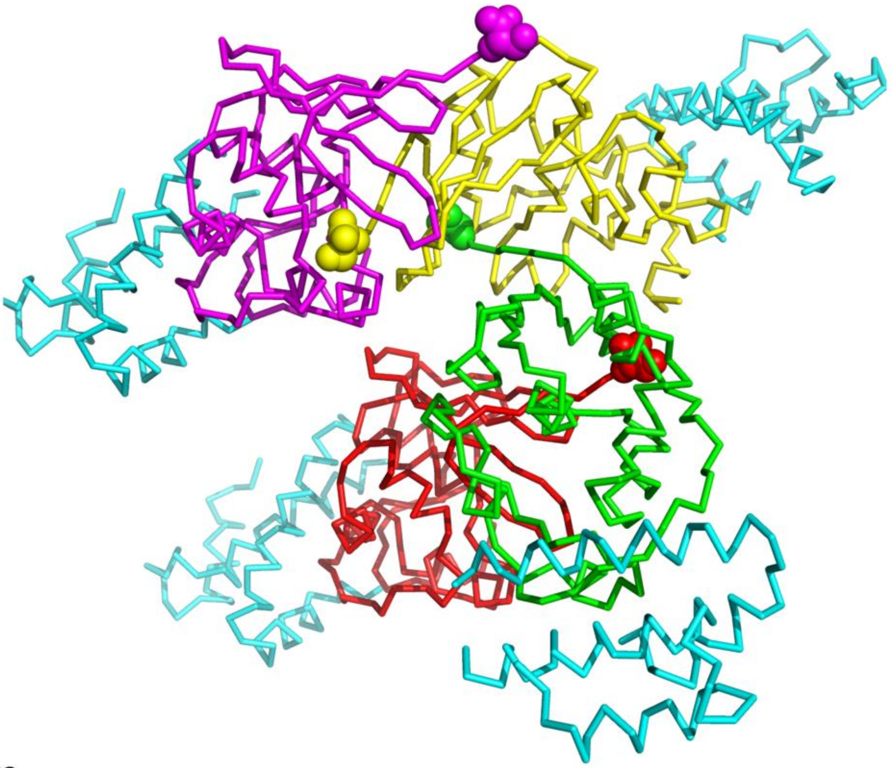

Figure S2

Figure S2: PRO makes up continuous helices in the crystal packing.

Four successive asymmetric units along the 3_1 crystallographic screw axis are shown as Cα traces. S15 molecules are in cyan and successive PRO molecules are red, green, yellow and magenta with the C-terminal serine 879 displayed as spheres. In the crystal, the parallel PRO helices are connected only through S15 dimers (not shown).

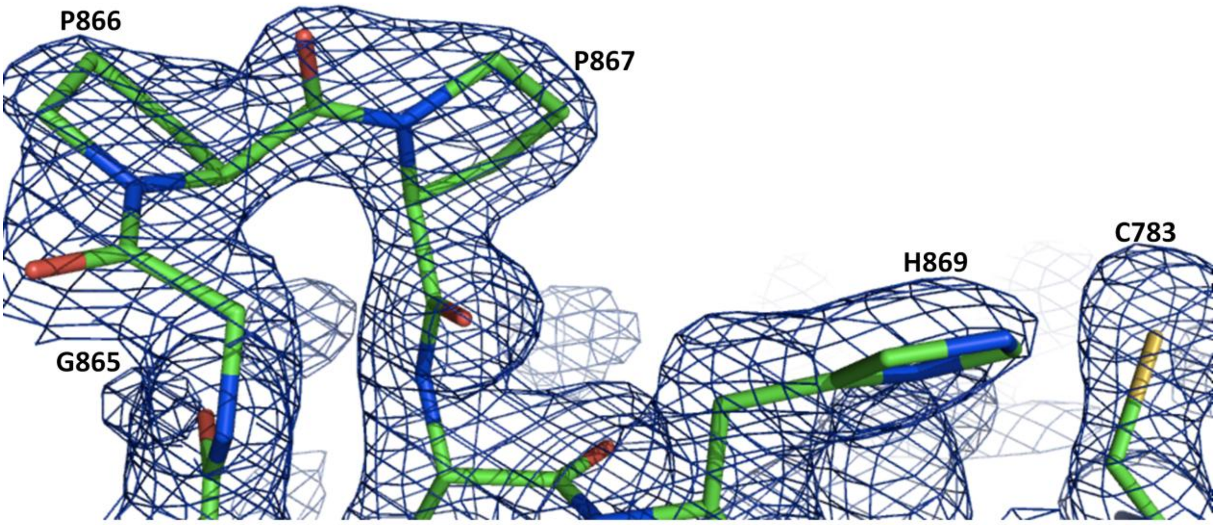

Figure S3

Figure S3: Two cis-prolines upstream of the catalytic histidine.

Final 2Fo-Fc electron density map for the two cis-prolines 865-Gly-Pro-Pro-867 directly upstream strand β6. Also visible and labeled is the catalytic dyad Cys783-His869.

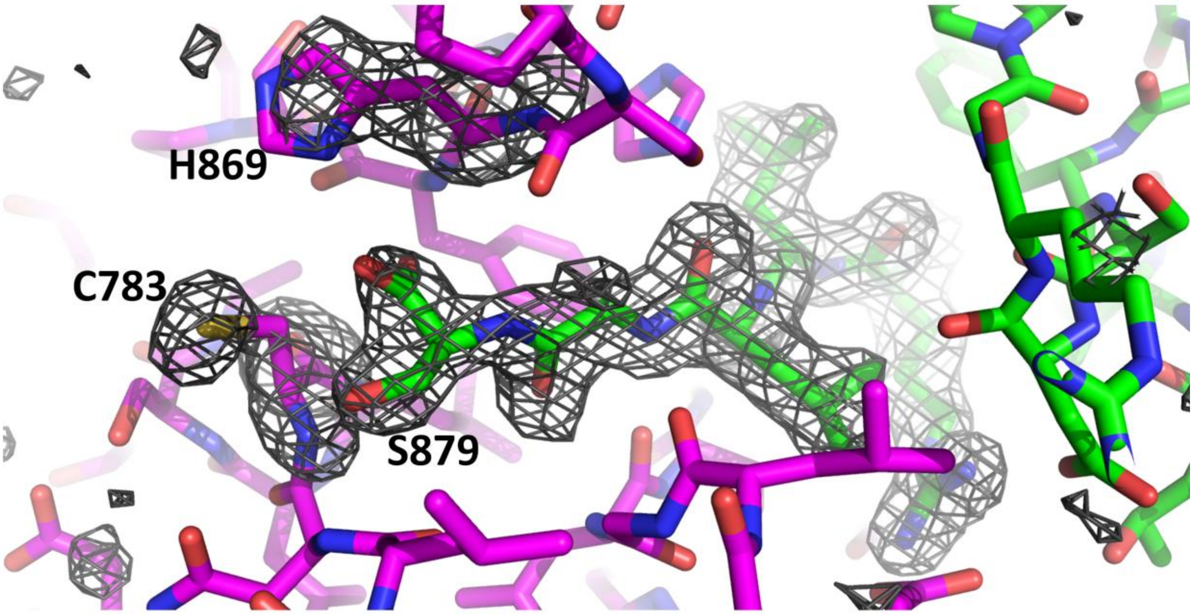

Figure S4

Figure S4: Simulated-annealing omit map after removal of the catalytic dyad and the C-terminal 5 residues.

The map is displayed at 3 sigma contour as a dark gray mesh. Superimposed is the final model (PDB 4a5u) as sticks with two PRO molecules displayed, the peptidase in magenta and the substrate in green. The catalytic dyad of the peptidase and the C-terminal serine of the substrate are labeled.

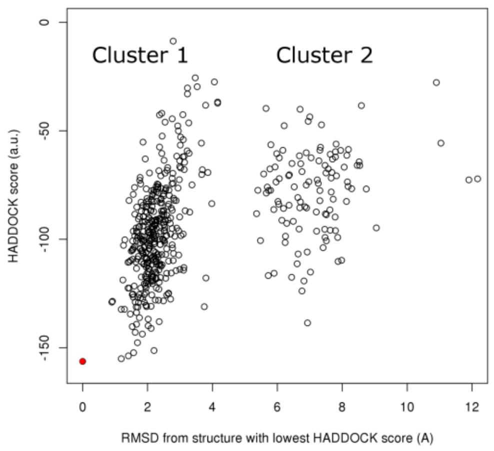

Figure S5

Figure S5: HADDOCK clustering analysis.

HADDOCK scores of the 500 refined structures docked using the HADDOCK software plotted against their interface backbone root mean square deviation (RMSD in Å) compared to the complex with the best HADDOCK score. The model selected and represented in Fig. 4CD is the complex with the lowest HADDOCK score (red filled dot) corresponding to the largest cluster and in good agreement with other docking methods (see Protocol S2).

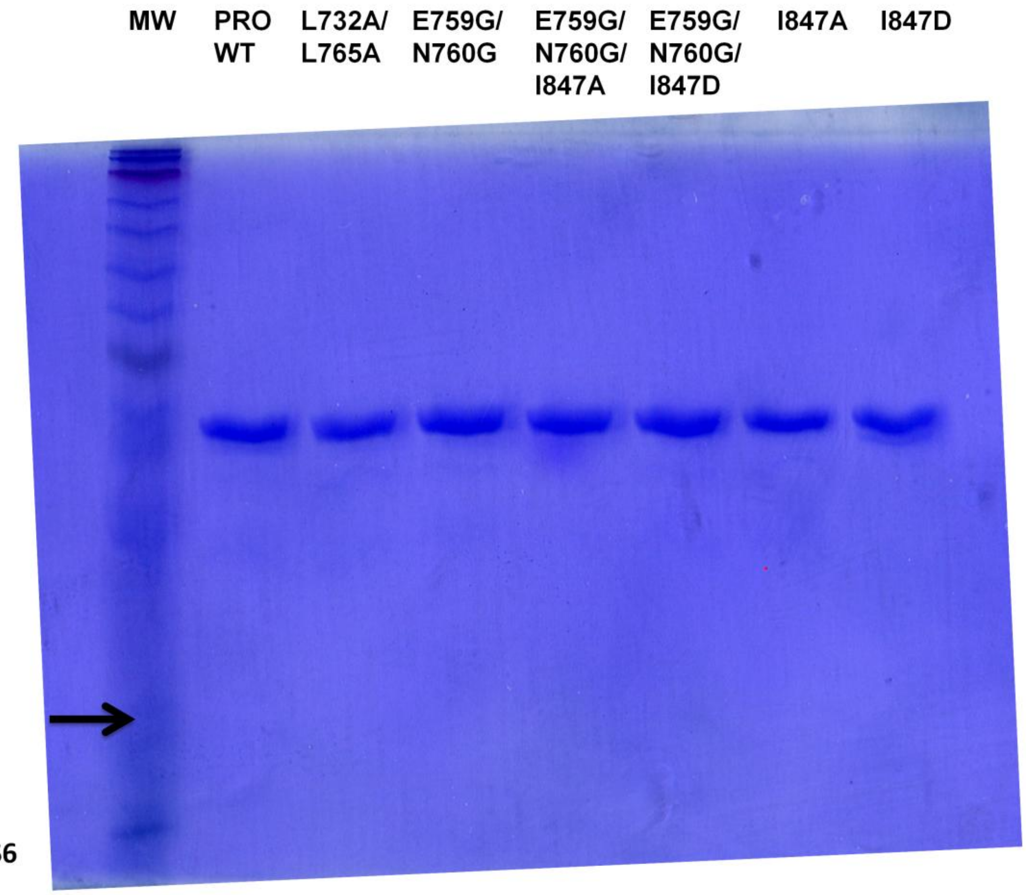

Figure S6

Figure S6: Coomassie-stained SDS-PAGE analysis of the PRO constructs used in the Ub-AMC deubiquitylation assays

From left to right: MW markers, PROWT, L732A/L765A, E759G/N760G, E759G/N760G/I847A, E759G/N760G/I847D, I847A, I847D.

1.5 µg of purified protein was deposited in each lane. The arrow indicates the expected position of S15.

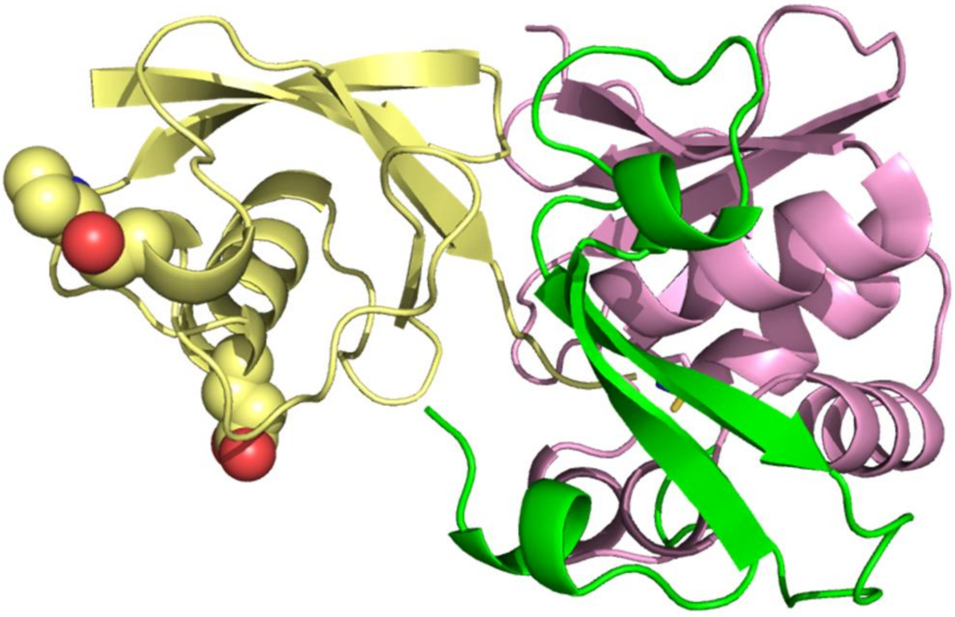

Figure S7

Figure S7: Location in the PRO/Ub docked model of ubiquitin residues not conserved in humans and plants

The model is displayed and colored as in Fig. 4C. The three residues not conserved between human and plant are drawn as spheres.
